# Cognitive and behavioural determinants of multiple sexual partnerships and condom use in South Africa: Results of a national survey

**DOI:** 10.4102/sajhivmed.v20i1.868

**Published:** 2019-06-10

**Authors:** Patience G. Manjengwa, Kerry Mangold, Alfred Musekiwa, Lazarus R. Kuonza

**Affiliations:** 1 South African Field Epidemiology Training Programme, National Institute of Communicable Diseases, Johannesburg, South Africa; 2 School of Health Systems and Public Health, University of Pretoria, Pretoria, South Africa; 3 South African National AIDS Council Trust, Pretoria, South Africa

**Keywords:** HIV, Multiple sexual partnerships, Non-condom use, Cognitive factor, Intergenerational sex, Perceived benefits, Perceived susceptibility, Personal beliefs

## Abstract

**Background:**

Human immunodeficiency virus (HIV) risky behaviours including multiple sexual partnership (MSP) and non-condom use (nCU) are known to be drivers of the spread of HIV; cognitive factors including perceived susceptibility of HIV, self-efficacy and attitudes play a significant role in influencing risky sexual behaviours.

**Objectives:**

We sought to investigate personal beliefs, perceptions, thoughts and actions that are associated with MSP and nCU in South Africa.

**Methods:**

We analysed nationally representative data from the 2012 National HIV Communication Survey (NCS) that included about 10 000 participants aged 16–55 years. Five constructs were created to measure psychosocial and cognitive determinants. Cronbach’s alpha coefficient for internal consistency reliability was calculated. Multivariable logistic regression was used to determine factors associated with MSP and nCU.

**Results:**

Of the 6061 sexually active respondents, 13% (95% CI: 11.47–13.12) reported MSP and 52.7% (*n* = 3158 of 6039) (95% CI: 51.0–53.55) nCU at last sex. Factors associated with MSP included perceived benefits, adjusted odds ratio (aOR) = 2.16 (95% CI: 1.80–2.58), perceived susceptibility to HIV, aOR = 2.22 (95% CI: 1.83–2.69) and engaging in intergenerational sex, aOR = 2.14 (95% CI: 1.78–2.56). Predictors of nCU were perceived benefits, aOR = 1.25 (95% CI: 1.09–1.43); perceived susceptibility to HIV, aOR = 1.6 (95% CI: 1.39–1.83); and personal beliefs, aOR = 1.35 (95% CI: 1.13–1.62).

**Conclusion:**

Cognitive and behavioural factors were found to be predictors of risky sexual behaviours for HIV. This highlights the importance of considering personal perception and reasoning when attempting to understand and influence an individual’s sexual behaviour. This could be done through enhancing awareness of HIV risk in the general population and by influencing cognitive behaviour change through community mobilisation, advocacy and creating activities to improve self-esteem.

## Introduction

Globally, human immunodeficiency virus (HIV) poses a major public health concern, causing high rates of mortality and morbidity.^1^ In 2013, there were 35.3 million people living with HIV, with approximately 2.3 m new HIV infections and more than 1.6 m HIV-related deaths.^2^ In sub-Saharan Africa, it was estimated that there were 23.5 m people living with HIV in March 2015.^2^ In 2012, HIV prevalence in South Africa (SA) among all age groups was 12.2%, an increase from 10.6% reported in 2008.^2^ With an HIV incidence rate of 4.5%, the increased prevalence of HIV in 2012 could be attributed to the combined effects of new infections and a successfully expanded antiretroviral treatment (ART) programme.^2^

Numerous societal, cultural and personal intrinsic factors have been identified as important social and structural drivers of the HIV epidemic in SA, including high population mobility and inequalities in wealth and gender.^3^ Other contributing drivers of HIV include attitudes and behaviours of men, intergenerational sex, gender and sexual violence, stigma and untreated viral sexually transmitted infections (STIs).^3,4,5,6,7^

SA is continuing to address social and structural factors that influence HIV and prevent new HIV infections.^2,6^ One of the goals of the South African National Strategic Plan on HIV, STIs and TB 2012–2016 is to reduce new HIV infections by at least 50%, using a combination of prevention approaches combining biomedical and behavioural interventions.^8^ In 2012, the South African government made a commitment to address issues related to social and structural factors that influence HIV through scaling up accessibility of services including ART, rolling out the HIV counselling and testing campaign, expanding medical male circumcision programmes and provision of basic needs grants.^6,7,9^ To reduce new HIV infections, a combination of biomedical, behavioural, social and structural interventions have been set in place and are being constantly improved for better alignment.^8^

Multiple and concurrent partnerships, low and inconsistent condom use, alcohol abuse (together termed risky sexual behaviours) and low levels of male circumcision have been shown to be the key drivers of the epidemic.^10,11^ While HIV risky behaviours are known to be drivers of the spread of HIV, cognitive factors including perceived susceptibility to HIV, perceived monetary or material benefits of having sex for material gain, self-efficacy and attitudes play a significant role in influencing risky sexual behaviours.^12,13,14^ Based on the health belief model, an individual’s personal belief influences their behaviour.^12^ Despite the large number of research studies carried out on risk factors of HIV in SA, which include age of sexual debut, age disparate or intergenerational relationships (5 year age difference), multiple sexual partnerships (MSPs) and condom use, there have been limited studies of cognitive behaviours that influence risky sexual behaviours. Cognitive behaviours include a perceived lack of susceptibility to HIV, perceived benefits, personal beliefs surrounding risky sexual behaviours, condom self-efficacy, social norms and the impact self-esteem has on engaging in risky sexual behaviours.^15^ A study conducted by Tarkang in Cameroon in 2013 revealed that only 39% of the sexually active secondary school learners had a high HIV risk perception.^16^ A study conducted by Pettifor et al. in SA reported that only 14.0% of school learners had high HIV risk perception.^17^ Perceptions, ideas and behaviours that determine people’s actions need to be explored further in order to better understand the drivers of risky sexual behaviours in SA.

There are a number of psychological concepts that show how ideational and cognitive factors can have an impact on behaviour modification. The acquired immune deficiency syndrome (AIDS) risk reduction model states that knowledge of HIV and AIDS is a prerequisite that will enable an individual to take action and change their behaviour. This model links HIV knowledge to behaviour change. However, findings regarding the correlation between knowledge and behaviour have been inconsistent.^3^ Other theories and models of health risk perception assert that cognitive ideational factors that are related to attitudes, beliefs, knowledge, intentions and perceived self-efficacy are sufficient to foster safer sex behaviour.^3,12^

This study sought to investigate personal beliefs, perceptions and other ideas, thoughts and actions that are associated with MSP and non-condom use (nCU) among the South African population aged 16–55 years.

## Methods

We analysed secondary data from the Third National HIV Communication Survey (NCS) conducted in all nine provinces in SA between February and May 2012. This survey was designed to be representative of 16–55 years old. The methodology has been previously published,^16^ but briefly a multi-stage, cluster sampling approach was first used to draw a sample of 400 primary sampling units. Secondly, a systematic sampling interval was calculated by probability proportional to size techniques. The third stage of the sampling focused on randomly selected households, followed by individuals.

## Measurements and variables

An interviewer-administered structured questionnaire was used to collect data, including socio-demographic characteristics, exposure to television and radio communication messages or programmes on HIV and AIDS, perception of risk and indicators on knowledge, attitude and behaviour related to HIV and AIDS.

Five constructs were created to measure psychosocial and cognitive determinants. Responses to the questions that made up the constructs were graded on a five-point Likert scale, ranging from strongly disagree to strongly agree. Cronbach’s alpha coefficient for internal consistency reliability was used to assess the correlations between the items that made up each construct. Values of 60% or higher were considered to indicate acceptable internal consistency. A composite score was obtained for each construct by calculating an average score of the responses to all the questions that made up the construct. We calculated the average scores in percentages. The composite score was used to create a dichotomous variable for the construct, which was graded as either high if the composite score was above 65% or low if the composite score was 65% or lower. This cut-off number was used to accommodate the small number of questions used on other constructs because we used questions from a survey that was intended to measure communication programmes in SA. Box 1 shows single-item questions that were used for each behavioural construct.

BOX 1 Single-item questions from the questionnaire of the National HIV Communication Survey 2012 used to create cognitive and social behaviour constructs.

**Multiple sexual partnership**

**Perception susceptibility: An individual’s view about HIV risk perception**

What do you think your chances are of getting infected with HIV

**Condom self-efficacy: A person’s feeling about ability to effectively use condoms**

Sometimes, in the morning after having sex without a condom, I think ‘my God, what did I do?’

I can use a condom even when I have too much to drink

I can refuse to have sex if someone I like refuses to use a condom

I can buy a condom without feeling embarrassed

[WOMEN] I am confident that I can correctly put a condom on a man when having sex with him

**Personal beliefs: An individual’s views about HIV-related issues**

If someone ever has trouble putting on a condom, they will be embarrassed to try to use a condom again

Men who use condoms with their wives are opening the door for her to have sex with other men

Using a condom will make your partner think you do not trust him or her

Now and then, I go to someone else besides my main partner because the sex is so good

It is ok to have sex with others as long as your main partner does not find out

**Perceived benefits: An individual’s perception of positive consequences brought by a certain behaviour**

When you use a condom, you cannot get enough pleasure

If you have good communication with your partner, you can be sexually satisfied with one person

I need someone else to fill the gap in case I ever break up with my main partner

Now and then, I go to someone else besides my main partner because the sex is so good

**Self-esteem: An individual’s confidence in his or her self-worth**

If someone has problems putting a condom, they will be ashamed to put it again

Using a condom will make your partner think you do not trust him or her

I feel that I am a person of worth, at least on an equal plane with others

I take a positive attitude towards myself

**Social capital and norms: How an individual thinks about HIV and other issues that affect people in their community**

People in my community take HIV and AIDS seriously

People in my community are joining together to help people with HIV and AIDS

If you wait to have sex, you will find the right person for yourself

When a relationship ends, you should wait a few months and do not rush into a sexual relationship

## Definitions

The perceived benefits construct was defined as beliefs that there are positive outcomes related to engaging in a specific behaviour. The self-efficacy construct was defined as beliefs that one is capable of completing a certain task on their own. Perceived susceptibility was defined as the individual’s belief that they would acquire HIV infection. Social norms are beliefs of how the society thinks people should perform or how the society views things. Personal beliefs are intrinsic cognitive beliefs that people have on their own. Multiple sexual partnerships (MSP) refers to having had more than one sexual partner in the past 12 months and nCU is defined as not using either a male or a female condom at last sex. Intergenerational sex was defined as having a sexual relationship with someone with a 5 year or more age difference.

## Statistical analysis

A descriptive cross-sectional analysis was conducted to describe the demographic and risk factors by age, sex and province. We used weighted data in our analysis to be representative of the SA population with respect to age, sex, province, population group and urban or rural residence. Sample weights were corroborated using the 2007 Community Survey conducted by Statistics South Africa. Chi-squared test was used to test for an association between the outcomes MSP and nCU and psychosocial and cognitive constructs. Univariate logistic regression was used to determine factors associated with the outcomes MSP and nCU. Manual forward stepwise procedure was used to select variables for the multivariable model. Multi-collinearity tests were performed and only non-collinear variables were analysed. Multivariable logistic regression was used to determine independent factors associated with outcome after adjusting for potential confounders such as sex, employment, age, relationship type, geography, settlement type, HIV status, condom use at last sex, intergenerational sex (difference in ages by 5 years) and alcohol use at last sex, perceived susceptibility, personal benefits, personal beliefs, social norms, self-esteem and condom self-efficacy. A *p*-value of less than 0.05 was considered statistically significant. All analyses were conducted using STATA 13.0 (Stata Corporation, College Station, TX, USA).

## Ethical consideration

All procedures performed in studies involving human participants were in accordance with ethical standards of the institutional and/or national research committee and with the 1964 Helsinki Declaration and its later amendments or comparable ethical standards. For this type of study, formal consent was not required.

## Results

The socio-demographic characteristics of the sampled population are shown in Table 1. Of the total sample of 10 034 participants, 6061 reported that they had at least one sexual encounter in the past 12 months. Of these sexually active participants, 41% (2467 of 6061) were men. The overall mean age was 31.3 years (s.d.: 11). Twenty-three per cent (1378 of 6061) of the participants were aged 20–24 years and 6% (371 of 6061) were aged 45–49 years. Overall, 39% (2374 of 6061) lived in urban formal settlements, 37% (2158 of 6061) were from urban informal settlements, 11% (659 of 6061) lived in peri-urban areas and only 2% (286 of 6061) lived in farming settlements. The majority of participants had some form of education, but 1% (56 of 6061) had no schooling. Participants with a high socio-economic status were 34.7% (2107 of 6061) and medium socio-economic status was almost similar at 38.5% (2335 of 6061).

**TABLE 1:** Background demographic characteristics of participants who had at least one sexual encounter in the past 12 months of the survey in the South African 16–55-year-old population, 2012.

Characteristics	*n* = 6061	%
**Sex**		
Women	3594	59.0
Men	2467	40.0
**Age**		
16–24	1799	30.0
25–49	3894	64.0
50–55	368	6.0
**Race**		
Black people	5029	82.0
Mixed race	824	14.4
White people	102	1.8
Indian people or Asian people	2	1.7
**Settlement**		
Urban formal	2374	39.0
Urban informal	2158	35.6
Peri-urban	659	10.87
Tribal settlement	712	11.75
Farming	158	2.61
**Socio-economic status**		
High	2107	34.7
Medium	2335	38.5
Low	1619	26.7
**Marital status**		
Single	1884	31.0
Not married, serious relationship	1516	25.0
Not married, living with partner	797	13.1
Married living with spouse	1425	23.5

## Demographic characteristics of respondents who reported multiple sexual partnership

A total of 13% (744/6061; 95% CI: 11.47–13.12) of the sexually active population aged 16–55 years reported having had MSP in the past 12 months. The mean age was 28 years (s.d. 7.62), with the majority (65%) being men (481/744). Table 3 shows that the majority (93%) were black people (696/744), 5% (41/744) were mixed race and less than 1% (7/744) were white people or Indians. Almost half had medium socio-economic status 45% (330/744). The highest percentage of people who engaged in MSP was recorded among single respondents 41% (306/744) followed by those not married or living together but in a steady relationship 33% (247/744).

**TABLE 2:** Demographic, HIV risk, cognitive and social predictors of multiple sexual partnership in the past 12 months among 16–55-year-old participants in South Africa, 2012.

MSP predictors	*n* = 744	%	Crude	95% CI	*p*	Adj	95% CI	*p*
**Sex**								
Men	481	64.65	3.06	2.61–3.6	< 0.001	-	-	-
Women	263	35.35	1	-	-	-	-	-
**Age**								
16–24	282	37.9	1	-	< 0.001	-	-	-
25–49	451	60.6	0.70	0.60–0.82	< 0.001	-	-	-
50–55	11	1.5	0.16	0.09–0.30	< 0.001	-	-	-
**Race**								
Black	696	93.6	1	-	-	-	-	-
Mixed races	41	5.5	0.32	0.23–0.45	< 0.001	-	-	-
White	4	0.5	0.25	0.09–0.69	0.007	-	-	-
Indians	3	0.4	0.18	0.05–0.58	0.004	-	-	-
**Marital status**								
Single	306	41.0	1.13	0.70–1.83	0.602	-	-	-
Not married or living together	247	33.2	1.14	0.70–1.84	0.594	2.1	1.07–4.11	0.03
Not married but living together	76	10.2	0.61	0.36–1.03	0.069	-	-	-
Married living with spouse	64	8.6	0.27	0.16–0.46	0.074	-	-	-
Married not living with spouse	17	2.3	0.53	0.27–1.06	0.897	-	-	-
Widowed	21	2.8	1	-	-	-	-	-
**Socio-economic status**								
Medium socio-economic status	330	44.3	1.42	1.18–1.71	< 0.001	1.24	1.01–1.55	0.05
Low socio-economic status	196	26.3	1.19	0.95–1.82	0.05	-	-	-
High socio-economic status	218	29.3	1	-	-	-	-	-
**Highest level of education**								
Up to Grade 11	312	41.9	1.37	1.00–1.89	0.05	1.72	1.13–2.63	0.01
Matric	267	35.9	1.32	0.95–1.82	0.093	1.7	1.11–2.62	0.014
Tertiary	107	14.4	1.45	1.01–2.08	0.04	2.04	1.27–3.28	0.003
No schooling	4	0.54	0.73	0.25–2.12	0.57	-	-	-
Primary school	48	6.5	1	-	-	-	-	-
**Perceived benefits**								
High	423	57.2	2.29	1.96–2.68	< 0.001	2.16	1.80–2.58	< 0.001
Low	316	42.7	1	-	-	-	-	-
**Perceived susceptibility**								
High	272	42.7	2.53	2.13–3.01	< 0.001	2.22	1.83–2.69	< 0.001
Low	365	57.3	1	-	-	-	-	-
**Transactional sex**								
Yes	199	26.8	5.97	4.89–7.29	< 0.001	-	-	-
No	545	73.2	1	-	-	-	-	-
**Alcohol use before sex**								
Yes	373	75.1	1.33	1.06–1.66	0.013	-	-	-
No	124	24.9	1	-	-	-	-	-
**Intergenerational sex**								
Yes	435	58.4	2.1	1.79–2.45	< 0.001	2.14	1.78–2.56	< 0.001
No	309	41.5	1	-	-	-	-	-
**Provinces**								
Eastern Cape	42	5.6	1.16	0.79–1.71	0.426	-	-	-
Free State	100	13.4	4.26	2.88–6.28	< 0.001	-	-	-
North West	61	8.2	2.63	1.73–3.99	< 0.001	2.4	1.48–3.89	< 0.001
Limpopo	39	5.2	1.38	0.92–2.08	0.116	-	-	-
Gauteng	225	30.2	2.54	1.77–3.63	< 0.001	-	-	-
KwaZulu-Natal	157	21.1	2.33	1.63–3.32	< 0.001	-	-	-
Mpumalanga	32	4.3	2.63	1.73–3.99	<0.001	-	-	-
Northern Cape	19	2.5	2.07	1.14–3.77	0.017	-	-	-
Western Cape	69	9.2	1	-	-	-	-	-

MSP, multiple sexual partnership; OR, odds ratio; CI, confidence interval.

**TABLE 3:** Demographic, HIV risk, cognitive and social predictors of non-condom use at last sex among sexually active 16–55-year-old participants in South Africa, 2012.

Non-condom use predictors	*n* = 3158	%	Crude		*p*	Adj		*p*
			OR	95% CI		OR	95% CI	
**Sex**								
Men	1202	38.1	0.79	0.72–0.88	< 0.001	-	-	-
Women	1956	61.9	1	-	-	-	-	-
**Age**								
16–19	421	7.0	1	-	-	1	-	-
20–24	1378	22.7	1.48	1.17–1.86	0.001	1.46	1.13–1.88	0.003
25–29	1294	21.4	1.95	1.54–2.46	< 0.001	1.72	1.33–2.22	< 0.001
30–34	995	16.4	2.75	2.16–3.24	< 0.001	2.28	1.74–3.00	< 0.001
35–39	718	11.8	2.90	2.25–3.74	< 0.001	1.96	1.46–2.63	< 0.001
40–44	516	8.5	3.57	2.72–4.68	< 0.001	2.12	1.54–2.92	< 0.001
45–49	371	6.1	5.24	3.86–7.10	< 0.001	3.03	2.11–4.34	< 0.001
50–55	368	6.0	8.89	6.39–12.97	< 0.001	5.25	3.54–7.77	< 0.001
**Race**								
Black people	2415	76.5	1	-	-	1	-	-
Mixed race	595	18.8	0.32	0.23–0.45	< 0.001	3.11	2.57–3.75	< 0.001
White people	73	2.3	0.25	0.09–0.69	0.007	2.12	1.29–3.49	0.003
Indian people	74	2.3	0.18	0.05–0.58	0.004	2.67	1.60–4.45	< 0.001
**Marital status**								
Single	700	22.0	0.53	0.37–0.74	0.001	0.94	0.62–1.43	0.790
Not married or living together	648	20.5	0.67	0.47–0.94	0.023	1.30	0.85–1.98	0.218
Not married but living together	476	15.1	1.33	0.93–1.90	0.069	2.04	1.32–3.14	0.001
Married living with spouse	1102	38.9	3.11	2.19–4.41	0.074	4.09	2.70–6.20	< 0.001
Married not living with spouse	123	3.9	1.41	0.91–2.17	0.897	1.88	1.13–3.12	0.014
Widowed	76	2.4	1	-	-	1	-	-
**Alcohol use before sex**								
Yes	859	73.1	1.27	1.07–1.52	0.006	-	-	-
No	315	26.9	1	-	-	-	-	-
**Settlement type**								
Urban formal	1211	37.1	1	-	0.998	1	-	-
Urban informal	1101	26.9	1.00	0.88–1.12	0.998	1.24	1.01–1.55	0.002
Peri-urban	361	35.9	1.16	0.97–1.38	0.090	1.28	1.04–1.57	0.017
Tribal settlement	381	12.0	1.10	0.93–1.30	0.265	1.59	1.30–1.94	< 0.001
Farming settlement	104	3.2	1.90	1.35–2.68	<0.001	2.15	1.46–3.15	< 0.001
**Personal beliefs**								
Yes	2522	80.5	1.38	1.21–1.59	< 0.001	1.35	1.13–1.62	0.001
No	610	19.4	1	-	-	-	-	-
**Perceived benefits**								
High	1302	41.6	1.22	1.10–1.35	< 0.001	1.25	1.09–1.43	0.001
Low	1824	58.4	1	-	-	-	-	-
**Perceived susceptibility**								
High	766	27.0	1.24	1.09–1.40	0.001	1.6	1.39–1.83	< 0.001
Low	2070	72.9	1	-	-	-	-	-

OR, odds ratio; CI, confidence interval.

## Demographic characteristics of respondents who reported non-condom use

Out of the total number of sexually active respondents, more than half reported not using a condom at last sex 53% (3158/6039, 95% CI: 51.03–53.55, *p* < 0.05). Mean age was 33 years (s.d. 10.02). Of the people who did not use condoms at last sex, 62% (1956/3158) were women. Twenty per cent of the people who reported nCU were from Gauteng province (GP) (632/3158) followed by Western Cape (WC) 18% (568/3158) and KwaZulu-Natal (KZN) 17% (562/3158). Among those with no schooling, 79% (44/56) did not use condoms at last sex. Non-condom use at last sex was common among those who had education up to Grade 11 (43%; 1355/3158). The prevalence of nCU among people with a high socio-economic status was 54% (1134/2097) and those with a low socio-economic status were 52% (852/1610). Of the people who reported not using condoms at last sex, married participants had the highest prevalence of nCU at last sex (35%; 1102/3158), followed by single (22%; 700/3158) and not married or living together but in a steady relationship (21%; 648/3158).

## Univariate analysis – Predictors of multiple sexual partnership

The odds of reporting MSP were two times higher among those engaging in intergenerational sex than those having sex with people in the same generation (OR 2.10, 95% CI: 1.79–2.46, *p* < 0.001). Participants who had consumed alcohol before sex were 1.3 times more likely to report MSP than those who did not consume alcohol before sex (OR 1.33, 95% CI: 1.06–1.67, *p* < 0.02). People who engaged in transactional sex were almost six times more likely to have MSP in the past 12 months than those who did not engage in transactional sex (OR 5.97, 95% CI: 4.89–7.29, *p* < 0.001). People who lived in Free State province were nearly four times more likely to report MSP (OR 3.73, 95% CI: 2.67–5.21, *p* < 0.001) than people living in WC province. Those living in GP were two times more likely to report engaging in MSP (OR 2.35, 95% CI: 1.74–3.09, *p* < 0.001) than people living in WC province. Students were almost 1.5 times likely to report having engaged in MSP than unemployed participants (OR 1.47, 95% CI: 1.13–1.89, *p* < 0.005).

## Univariate analysis – Predictors of non-condom use at last sex

Employed participants were 1.17 times (OR 1.17, 95% CI: 1.05–1.31, *p* < 0.005) more likely to report to have not used condoms at last sex than their unemployed counterparts. People living in farming settlements were almost two times more likely to have not used a condom at last sex than those living in urban formal (OR 1.91, 95% CI: 1.35–2.68, *p* < 0.001). Drinking alcohol in the past month was significantly associated with nCU at last sex (OR 1.27, 95% CI: 1.07–1.53, *p* < 0.001).

## Multivariable analysis – Predictors of multiple sexual partnership and non-condom use

The multivariable analysis showed that perceived benefits (aOR 2.16, 95% CI: 1.80–2.58, *p* < 0.001) and low perceived susceptibility (aOR 2.22, 95% CI: 1.83–2.69, *p* < 0.001) to HIV infection were the two psychosocial and cognitive constructs that were significantly associated with MSP. Intergenerational sex (aOR 2.14, 95% CI: 1.78–2.56, *p* < 0.001), medium socio-economic status (aOR 1.24, 95% CI: 1.01–1.55, *p* = 0.05) and having tertiary education (aOR 2.04, 95% CI: 1.27–3.28, *p* < 0.005) were additional predictors of MSP. Results in Table 1 show that after adjusting for confounders, personal belief around condoms (aOR 1.35, 95% CI: 1.13–1.62, *p* < 0.005), high perceived benefits (aOR 1.25, 95% CI: 1.09–1.43, *p* < 0.005) and low perceived susceptibility to HIV infection (aOR 1.6, 95% CI: 1.39–1.83, *p* < 0.001) were identified as psychosocial and cognitive factors that influence nCU at last sex. The final multivariable model for nCU, Table 3, retained living in farming settlements (aOR 2.15, 95% CI: 1.46–3.15, *p* < 0.001) and age group (30–34) compared to 16–19 year olds (aOR 2.28, 95% CI: 1.74–3.01, *p* < 0.001) together with the psychosocial and cognitive factors mentioned above.

## Discussion

In this study, low perceived susceptibility of HIV infection and perceived monetary, material or cognitive benefits were significantly associated with both MSP and nCU at last sex. Similar associations were found in a study conducted in Cameroon in 2012, which revealed the association of MSP and low-risk perception of HIV infection.^16^ It is very concerning to note that people perceive themselves to be at lower risk of acquiring HIV infection despite engaging in risky sexual behaviour. It is generally known that people judge a potential threat through their past experiences and anticipated consequences.^12,18^ Low perceived susceptibility could be partially attributed to the fact that HIV and AIDS is a highly stigmatised disease. Therefore, when a person acknowledges his or her risk of acquiring HIV infection, he or she becomes vulnerable to being stigmatised. Because of this risk, people may avoid self-disclosure and by so doing downplay their personal risk.

A high proportion of black participants reported engaging in MSP, followed by mixed race participants as compared to the Indian participants. Multiple sexual partnership was significantly higher among men than among women. This is consistent with other studies where generally wealthy men in patriarchal societies like SA are expected to have numerous partners or wives. This follows the polygamous culture in African countries.^19^ Furthermore, women have been found generally to under-report sexual behaviours.^8,20^ This can be explained by the fact that women want their manner to be viewed favourably by others. MSP in women is often viewed in a derogatory sense.

The proportion of respondents who had reported not using condoms at last sex was 57%. This was higher among women (62%) than among men (38%). The figure appeared similar to the findings of the South African National HIV Prevalence, Incidence and Behaviour Survey, 2012, which reported nCU as 63% in the whole population.^2^ Engaging in MSP and having unprotected sex increases HIV risk because of the fact that individuals may be linked through sexual networks and become more vulnerable to high viral load exposure during the early phases of new HIV infection.^21^

Low socio-economic status was predictive of nCU at last sex but there was a significant association with MSP. In this study, MSP was frequently reported among those with medium socio-economic status and least reported among respondents with a high socio-economic status. Similarly, according to a survey conducted on young men and women in SA in 2004, MSP was reported least among those with high socio-economic status. An explanation of a similar finding suggested that if people with lower socio-economic status are compared to the ones with a high socio-economic status, those with lower socio-economic status may choose to spend more of their income pursuing various forms of relationships.^2,5^ Another possible explanation could be that respondents in the high socio-economic group may have higher educational attainment and better health information; hence, they reduce risky sexual behaviours.^22,23^

MSP was prevalent among participants who engaged in intergenerational sex. It has been shown in this study and other studies that intergenerational sex fuels the HIV epidemic among the younger generations.^19,24^ Research studies argue that when young women mix with the older generation, their risk for contracting HIV increases.^2,25^ Interventions targeted at reducing intergenerational sex could reduce the prevalence of HIV among younger generations. It is concerning that the highest proportion of MSP is among the 20–24-year age groups where both boys and girls are affected. Age mixing with older generations further exacerbates this problem because young girls are likely to mix with both older men and young boys.^26^ In this case, old men infect young girls who then infect young boys. Studies have confirmed that sex with older men is more risky than sex with younger men because HIV prevalence among older men is significantly higher than younger men.^24^ The age differential with older men also introduces a power dynamic into the sexual relationship where younger women are more vulnerable and less likely to successfully negotiate condom use. Intergenerational sex is also closely entwined with transactional sex – where economic factors push young girls to engage in various forms of sex in exchange for cash or material benefits.^1^

It is not surprising then that transactional sex was also associated with MSP. Almost half of the participants reporting both MSP and transactional sex were among those with medium socio-economic status. This implies that these participants were not poor but rather alludes to need for economic gains or advancements and wealth inequalities as a push factor towards engaging in HIV risk behaviours. The need for social upward mobility could explain the need for participants to engage in MSP and transactional sex.^3^

Increased HIV incidence has been associated with low socio-economic status in recent studies.^1,20^ However, the practice of MSP is not untouched by employment status. With regard to employment status, students recorded a higher prevalence of MSP than the unemployed category. This finding is in contradiction to another study conducted in SA that showed that nearly half of the participants who engaged in MSP were unemployed and only 10% were students.^24^ These differences in findings could be because of the differences in how the studies defined unemployed group. This study considered informal employment as employed, while the other study categorised it as unemployed. The geographical coverage of the two studies could also explain the different findings. This analysis utilised data from a national survey, whereas the other study referred to was only undertaken in two provinces in SA. Further research will need to be undertaken to confirm these findings.

The high prevalence of non-condom use is exacerbated by the consumption of alcohol.^1,9,27^ Alcohol has adverse side effects that include sexual risk behaviours and these have been documented in various different studies in SA.^1^ Poor judgement and risky sexual behaviours are often exacerbated as a result of alcohol consumption as it impairs judgement and reduces inhibition. These findings emphasise the risks associated with the mix of sexual risk behaviours, MSP, transactional sex and alcohol; therefore, interventions better equipped for the complexities of the behaviour mix need to be put in place.

Perceived material, monetary or cognitive benefits were strongly associated with nCU and this was furthermore compounded by the association found between nCU, engaging in transactional sex and having MSP. While perceived benefits could be judged by anticipated rewards, in this study, there was an association with both nCU and MSP.^28^ Among those who did not use condoms at last sex, perceived benefits were significantly high. Similarly, other studies have found that people engage in a mix of MSP, nCU and transactional sex to access a fashionable lifestyle.^19,24^ This may be also explained why we found that students were more likely to engage in risky sexual behaviours as they are vulnerable to peer pressure and living up to a standard. The findings of this research showed that the participants did not use condoms because of their perception of benefits acquired. They perceived that not wearing condoms will make them get more money as compared to wearing condoms and the risk for HIV infection.

The findings of nCU after adjusting for confounders of transactional sex, alcohol use before sex and MSPs revealed that personal beliefs had an impact on condom use. Participants believed that it was unpleasurable to use condoms. This is consistent with other studies that have revealed that attitudes about condoms are predictive of condom use.^29^ While marital status strongly correlated with nCU and remained stronger after adjusting for confounders, being married was significantly associated with nCU; this is consistent with most studies. This finding is concerning and people in all types of relationships should be encouraged to use condoms, especially in a country where MSP is common practice regardless of marital status.

Living in a rural area or farming settlement was found to be a risk factor for not using condoms at last sex. This could be because of stigma and patriarchal norms which play a larger role in determining behaviour.^30^ This could also be because of logistical challenges of condom supply because of these areas being in difficult to reach or sparse areas of the country.

In our analysis, we had some limitations which included the fact that data used in this study analysis relied on self-reported sexual behaviour on sensitive issues, such as condom use and HIV and AIDS. Self-reported data are prone to social desirability bias where respondents tend to respond to questions in a manner that is viewed favourably by others. There is the possibility that participants could have exaggerated behaviour or under-reported undesirable behaviour. It is, however, unlikely that this bias affected our results because assurance of confidentiality and anonymity was given and the questionnaire was administered in a consistent manner across the whole sample. A further limitation is that the survey was cross-sectional in nature, and hence causality was difficult to establish. To overcome this challenge, we only reported on associations and correlations.

## Conclusion and recommendations

Our study analysed determinants of MSP and nCU and revealed that a low perceived susceptibility to HIV infection and that a high perception of benefit are common cognitive constructs correlated strongly to risky behaviours. Our results highlight the need to expand on several initiatives including prevention efforts and changing cognitive and psychosocial thinking. Firstly, HIV prevention efforts could be performed through encouraging avoidance of extramarital sex and the importance of condom use in all types of relationships, especially where high-risk sexual behaviour takes place such as MSP. Secondly, these results show that initiatives need to focus more closely on changing cognitive and psychosocial thinking in terms of personal beliefs and norms including the constructs of perceived benefits and perceived susceptibility. This could be done through enhancing awareness of HIV risk in the general population and other cognitive behaviour change interventions. Therefore, community mobilisation, advocacy, creating activities to improve self-esteem and aim to increase risk perception are of paramount importance.

Multi-sectorial efforts focusing on the social and structural drivers of risky sexual behaviours and HIV need to be prioritised. This includes psychosocial, health, educational and economic interventions. Lastly, the findings of this research will contribute to the knowledge about personal intrinsic factors and the psychosocial factors that predispose people to engage in risky sexual behaviours and help close a literature gap in understanding the dynamics of the epidemic. Cognitive factors must be prioritised and explored further in terms of the roles they play in HIV incidence.

## References

[1] Johnson SKD, Figueroa ME, Delate R, Mahlasela L, Magni S The Third National HIV Communication Survey 2012. 2013 https://www.ccisa.org.za/sites/default/files/hiv_survey.pdf

[2] Simbayi LC, Shisana O, Rehle T, et al South African national HIV prevalence, incidence and behaviour survey, 2012. Pretoria Hum Sci Res Counc [serial online]. 2014 [cited 2015 Aug 30]; Available from: http://www.hsrc.ac.za/en/research-outputs/view/6871

[3] WHO, UNAIDS, UNICEF Global HIV/AIDS response: Epidemic update and health sector progress towards universal access: Progress report 2011. Geneva: WHO, 2011; p. 233.

[4] Leclerc-Madlala S What really drives HIV/AIDS in Southern Africa? Summary report of SADC expert think-tank meeting in Maseru, 10–12 May 2006. AIDS Leg Q Netw Newsl. 2006:29–32.

[5] Hallett TB, Gregson S, Lewis JJC, Lopman BA, Garnett GP Behaviour change in generalised HIV epidemics: Impact of reducing cross-generational sex and delaying age at sexual debut. Sex Transm Infect. 2007;83 (Suppl 1):i50–i54. 10.1136/sti.2006.023606 17314125

[6] [PDF] from researchgate.net [homepage on the Internet] [cited 2015 Aug 19]. Available from: http://www.researchgate.net/profile/Michelle_Kaufman/publication/6539730_Alcohol_Use_and_Sexual_Risks_for_HIVAIDS_in_Sub-Saharan_Africa_Systematic_Review_of_Empirical_Findings/links/00b4952dfdb677c67e000000.pdf

[7] Mah TL, Halperin DT Concurrent sexual partnerships and the HIV epidemics in Africa: Evidence to move forward. AIDS Behav. 2008;14(1):11–16. 10.1007/s10461-008-9433-x 18648926

[8] Halperin DT, Epstein H Why is HIV prevalence so severe in southern Africa?: The role of multiple concurrent partnerships and lack of male circumcision-implications for HIV prevention: Opinion. South Afr J HIV Med. 2007;(26):19–23. 10.4102/sajhivmed.v8i1.630

[9] Council SANA National strategic plan on HIV, STIs and TB 2012–2016. Pretoria: SANAC; 2011.

[10] Venkataramani AS, Maughan-Brown B, Nattrass N, Ruger JP Social grants, welfare, and the incentive to trade-off health for income among individuals on HAART in South Africa. AIDS Behav. 2010;14(6):1393–1400. 10.1007/s10461-009-9642-y 20033277

[11] Greif MJ, Dodoo FN-A, Jayaraman A Urbanisation, Poverty and Sexual Behaviour: The Tale of Five African Cities. Urban Studies. 2010;48(5):947–57. 10.1177/0042098010368575 21744541

[12] [PDF] from ajol.info [homepage on the Internet] [cited 2015 Aug 19]. Available from: http://www.ajol.info/index.php/ajrh/article/viewFile/109250/99030

[13] Baliunas D, Rehm J, Irving H, Shuper P Alcohol consumption and risk of incident human immunodeficiency virus infection: A meta-analysis. Int J Public Health. 2010;55(3):159–166. 10.1007/s00038-009-0095-x 19949966

[14] Rosenstock IM, Strecher VJ, Becker MH The health belief model and HIV risk behavior change In DiClemente R. J. & Peterson J. L. (eds.), AIDS prevention and mental health. Preventing AIDS: Theories and methods of behavioral interventions (pp. 5–24). New York, NY, US: Plenum Press 1994 10.1007/978-1-4899-1193-3_2

[15] Godin G, Kok G The theory of planned behavior: A review of its applications to health-related behaviors. Am J Health Promot. 1996;11(2):87–98. 10.4278/0890-1171-11.2.87 10163601

[16] Fishbein M The role of theory in HIV prevention. AIDS Care. 2000;12(3):273–278. 10.1080/09540120050042918 10928203

[17] Prata N, Morris L, Mazive E, Vahidnia F, Stehr M Relationship between HIV risk perception and condom use: Evidence from a population-based survey in Mozambique. Int Fam Plan Perspect. 2006;192–200. 10.1363/3219206 17237016

[18] Tarkang EE Factors associated with perception of risk of contracting HIV among secondary school female learners in Mbonge subdivision of rural Cameroon. Pan Afr Med J [serial online]. 2014 [cited 2015 Aug 30];17. Available from: http://www.ncbi.nlm.nih.gov/pmc/articles/PMC4189865/10.11604/pamj.2014.17.259.2772 PMC4189865 25309659

[19] HIV and sexual behaviour among young South Africans: A national survey of 15–24 year olds | POPLINE.org [homepage on the Internet]. [cited 2015 Aug 30]. Available from: http://www.popline.org/node/253955

[20] [PDF] from researchgate.net [homepage on the Internet] [cited 2015 Aug 19]. Available from: http://www.researchgate.net/profile/Gaston_Godin/publication/13133717_The_theory_of_planned_behavior_a_review_of_its_applications_to_health-related_behaviors/links/0046352408c7d0be88000000.pdf

[21] Negeri EL Determinants of risky sexual behavior, relation between HIV risk perception and condom utilization among Wollega University Students in Nekemte Town, Western Ethiopia. Sci Technol Arts Res J. 2014;3(3):75–86. 10.4314/star.v3i3.13

[22] Shisana O, Rehle T, Simbayi LC, Zuma K, Jooste S South African national HIV prevalence incidence behaviour and communication survey 2008: A turning tide among teenagers? [serial online] 2009 [cited 2015 Aug 19]. Available from: http://www.popline.org/node/206526

[23] Raj A, Reed E, Miller E, Decker MR, Rothman EF, Silverman JG Contexts of condom use and non-condom use among young adolescent male perpetrators of dating violence. AIDS Care. 2007;19(8):970–973. 10.1080/09540120701335246 17851992

[24] Sh K Determinants of multiple sexual partners and condom use among sexually active Tanzanians. East Afr Med J. 1996;73(7):435–442. 8918004

[25] Nshindano C, Maharaj P Reasons for multiple sexual partnerships: Perspectives of young people in Zambia. Afr J AIDS Res. 2008;7(1):37–44. 10.2989/AJAR.2008.7.1.5.433 25871270

[26] Leclerc-Madlala S Age-disparate and intergenerational sex in southern Africa: The dynamics of hypervulnerability: AIDS. 2008;22(Suppl 4):S17–S25. 10.1097/01.aids.0000341774.86500.53 19033752

[27] Morojele NK, Kachieng’a MA, Mokoko E, et al Alcohol use and sexual behaviour among risky drinkers and bar and shebeen patrons in Gauteng province, South Africa. Soc Sci Med. 2006;62(1):217–227. 10.1016/j.socscimed.2005.05.031 16054281

[28] Cooper ML Alcohol use and risky sexual behavior among college students and youth: Evaluating the evidence. J Stud Alcohol Suppl. 2002;14:101–117. 10.15288/jsas.2002.s14.101 12022716

[29] Noar SM Behavioral interventions to reduce HIV-related sexual risk behavior: Review and synthesis of meta-analytic evidence. AIDS Behav. 2008;12(3):335–353. 10.1007/s10461-007-9313-9 17896176

[30] Ghimire L, Smith WCS, Van Teijlingen ER, Dahal R, Luitel NP Reasons for non-use of condoms and self-efficacy among female sex workers: A qualitative study in Nepal. BMC Womens Health. 2011;11(1):42 10.1186/1472-6874-11-42 21943102 PMC3206429

